# An Enterotoxin-Like Binary Protein from Pseudomonas protegens with Potent Nematicidal Activity

**DOI:** 10.1128/AEM.00942-17

**Published:** 2017-09-15

**Authors:** Jun-Zhi Wei, Daniel L. Siehl, Zhenglin Hou, Barbara Rosen, Jarred Oral, Christopher G. Taylor, Gusui Wu

**Affiliations:** a DuPont Pioneer, Hayward, California, USA; b DuPont Pioneer, Johnston, Iowa, USA; c Department of Plant Pathology, Ohio State University, Columbus, Ohio, USA; North Carolina State University

**Keywords:** Pseudomonas protegens, nematode, anti-nematode protein, binary toxin, enterotoxin, enterotoxins

## Abstract

Soil microbes are a major food source for free-living soil nematodes. It is known that certain soil bacteria have evolved systems to combat predation. We identified the nematode-antagonistic Pseudomonas protegens strain 15G2 from screening of microbes. Through protein purification we identified a binary protein, designated Pp-ANP, which is responsible for the nematicidal activity. This binary protein inhibits Caenorhabditis elegans growth and development by arresting larvae at the L1 stage and killing older-staged worms. The two subunits, Pp-ANP1a and Pp-ANP2a, are active when reconstituted from separate expression in Escherichia coli. The binary toxin also shows strong nematicidal activity against three other free-living nematodes (Pristionchus pacificus, Panagrellus redivivus, and Acrobeloides sp.), but we did not find any activity against insects and fungi under test conditions, indicating specificity for nematodes. Pp-ANP1a has no significant identity to any known proteins, while Pp-ANP2a shows ∼30% identity to E. coli heat-labile enterotoxin (LT) subunit A and cholera toxin (CT) subunit A. Protein modeling indicates that Pp-ANP2a is structurally similar to CT/LT and likely acts as an ADP-ribosyltransferase. Despite the similarity, Pp-ANP shows several characteristics distinct from CT/LT toxins. Our results indicate that Pp-ANP is a new enterotoxin-like binary toxin with potent and specific activity to nematodes. The potency and specificity of Pp-ANP suggest applications in controlling parasitic nematodes and open an avenue for further research on its mechanism of action and role in bacterium-nematode interaction.

**IMPORTANCE** This study reports the discovery of a new enterotoxin-like binary protein, Pp-ANP, from a Pseudomonas protegens strain. Pp-ANP shows strong nematicidal activity against Caenorhabditis elegans larvae and older-staged worms. It also shows strong activity on other free-living nematodes (Pristionchus pacificus, Panagrellus redivivus, and Acrobeloides sp.). The two subunits, Pp-ANP1a and Pp-ANP2a, can be expressed separately and reconstituted to form the active complex. Pp-ANP shows some distinct characteristics compared with other toxins, including Escherichia coli enterotoxin and cholera toxin. The present study indicates that Pp-ANP is a novel binary toxin and that it has potential applications in controlling parasitic nematodes and in studying toxin-host interaction.

## INTRODUCTION

Bacteria and nematodes are ubiquitous soil organisms and are subject to coevolutionary pressure. Many nematode species feed on bacteria and certain soil bacteria have evolved systems to combat predation from nematodes. Indeed, many microbial antagonists of nematodes have been found in nature. Some pathogenic bacteria are capable of proliferating in and killing nematodes by an infectious process or through the use of toxins (1–4). Some rhizosphere bacteria, such as Pseudomonas and Serratia, showed antagonistic effects on plant parasitic nematodes by proteinases and secondary metabolites (5–8). Control of plant-parasitic nematodes has been attempted through the use of biological organisms, which are typically “natural predators” of the nematode species (5, 7). The mechanisms of antagonism are yet to be fully understood. Isolating active compounds or proteins from such bacteria and analyzing their functions will help elucidate biocontrol mechanisms and promote their application in controlling plant-parasitic nematodes, which are major pests of some economically important row crops, vegetables, and fruit trees, and cause severe plant yield reduction worldwide (9, 10).

In this study, we screened diverse soil microbes for nematicidal activity against the free-living nematode Caenorhabditis elegans. From one potent strain, Pseudomonas protegens 15G2, we identified a novel binary toxin, designated Pp-ANP, which showed strong nematicidal activity against multiple nematode species. This toxin holds promise for controlling parasitic nematodes and is useful for studying toxin-host and bacterium-nematode interactions.

## RESULTS

### Pseudomonas protegens 15G2 shows strong anti-nematode activity.

**FIG 1:**
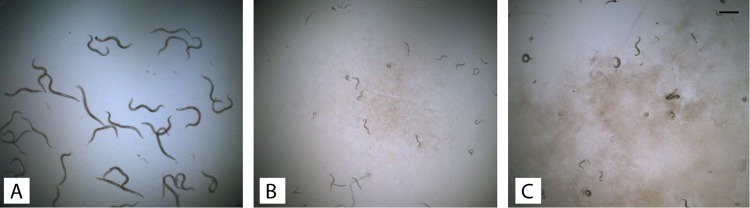
Inhibitory effect of P. protegens 15G2 strain on C. elegans after 2 days with L1 larvae. (A) Feeding with E. coli OP50 culture. (B) Feeding with 15G2 culture mixed with E. coli OP50. (C) Feeding with 15G2 crude protein mixed with E. coli OP50. Bar = 250 μm.

Various bacterial strains were isolated from soil samples collected throughout the United States. Overnight cultures of individual bacterial strains were tested against C. elegans in a liquid assay. Multiple experiments and multiple repeats for each strain were carried out to minimize variation. Some strains retarded the growth of C. elegans, while several strains completely inhibited development by arresting worms at the L1 stage, indicating nematicidal activity. Strain 15G2, isolated from a Missouri sample (11), showed strong activity and was selected for further investigation. The 15G2 soluble protein (clear lysate) showed strong activity against C. elegans (Fig. 1), but no activity was detected when the cell lysate was treated with heat (99°C, 20 min) or protease K (50°C, 30 min), suggesting that the activity was proteinaceous.

To identify strain 15G2, genomic DNA was isolated from the cell pellet and two specific PCR primers (AGAGTTTGATCMTGGCTCAG and TACCTTGTTACGACTT) were used to amplify an rRNA gene fragment. A BLAST search of the resultant 1,408-bp PCR product against the NCBI database revealed that 15G2 is Pseudomonas protegens.

### Two proteins are responsible for the nematicidal activity.

**FIG 2:**
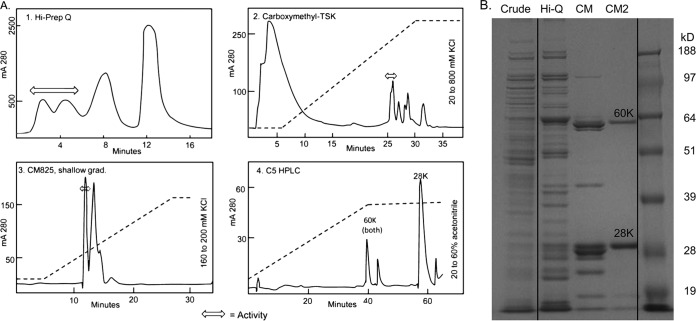
Separation and purification of nematicidal activity from 15G2. (A) Separation profiles. Arrow bars show active fractions. (B) SDS-PAGE of crude lysate and active fractions after Hi-Prep Q (Hi-Q) and carboxymethyl 825 separations (CM, CM2). Lines show where extraneous lanes were digitally excised from an image of a single gel.

To identify the active protein(s) from the total protein sample, the clear lysate was fractionated by column chromatography and activity was tracked with the C. elegans bioassay. The activity did not bind to the anion exchanger Hi-Prep Q at pH 7.2, but 3-fold enrichment was attained in the flowthrough. After concentrating and gel-filtering into buffer at pH 6.9, the material was applied to a sulfopropyl cation exchanger and eluted with a linear gradient to 200 mM KCl. Further purification was attained with two rounds of a lower capacity but higher resolution carboxymethyl cation exchanger, CM825. The anti-nematode activity was eluted within a shallow gradient of 160 to 200 mM KCl in 25 mM HEPES, pH 6.9 (Fig. 2A). At this stage, the purification was 2,280-fold with about 30% recovery (Table 1). Proteins in the active fractions had been reduced to four visible bands on SDS-PAGE, two major bands at 60 kDa and 28 kDa and two minor bands at 20 kDa and 14 kDa (Fig. 2B). Gel filtration chromatography on Superdex 75 confirmed that the 60-kDa protein was associated with the activity. The 60-kDa protein was designated Pp-ANP (*P*seudomonas *p**rotegens* antinematode protein). The band was excised from an SDS-PAGE gel and subjected to N-terminal sequencing. Based on the relative abundance of amino acids in each cycle of the Edman degradation, the results indicated that the band comprised two different proteins.

**TABLE 1:** Purification of anti-nematode protein from P. protegens 15G2

Stage	Protein^*a*^ (mg)	Activity^*b*^ for GI80 (μg)	Total activity (no. GI80 doses)	Purification fold	Recovery % of crude
Crude	363	121	3000		
Concentrated Q	40.4	14.1	2857	8.56	95.2
CM825	1.51	0.89	1700	136	56.7
2nd CM825	0.0485	0.0548	908	2280	30.3

a Protein concentrations are measured by the Bradford method with a bovine serum albumin (BSA) standard. Each 60K protein is ∼10% of total protein after the second CM825 experiment; therefore, GI80 dose is ∼5.5 ng.

b GI80 is the dose required for 80% growth inhibition, obtained from testing a range of dilutions in the bioassay.

Fractions corresponding to the 60-kDa band were pooled and resolved by reverse-phase (RP) high-pressure liquid chromatography (HPLC). Two separate peaks (Peak-1 and Peak-2) were observed in the separation profile (Fig. 2A, panel 4). The two peaks, singly and together, showed no anti-nematode activity in the bioassay, indicating protein denaturation during the RP-HPLC process. Edman sequencing was applied to the two separated samples to obtain the N-terminal amino acid sequences. The samples were also treated with trypsin and the tryptic peptides were sequenced to obtain internal peptide sequences. High quality N-terminal and several internal sequences were obtained for both proteins (Table 2).

**TABLE 2:** Peptide sequences obtained with Edman sequencing of Peak-1 and Peak-2 samples

Sample	N-terminal sequencing results	Internal sequencing results
Peak-1	APVNNPSGSYSGVHTPYVGY	AAYSGALTALMAGSSLWINCSGK
		ADSPWAK
		SSNEYWLSNYK
Peak-2	DNLPNVYRAVGLLDT	ESSESYGEEFFYK
		YDSGYVATTTLR
		AGGFFPK
		AVGLLXTPAAT

### Pp-ANP is encoded by two neighboring open reading frames.

Multiple degenerate primers were designed based on the peptide sequences and various PCR conditions were tested with 15G2 genomic DNA as the template. A 199 bp DNA fragment was obtained and it encoded part of the N-terminal and all three internal peptide sequences from the Peak-1 protein sample.

**FIG 3:**
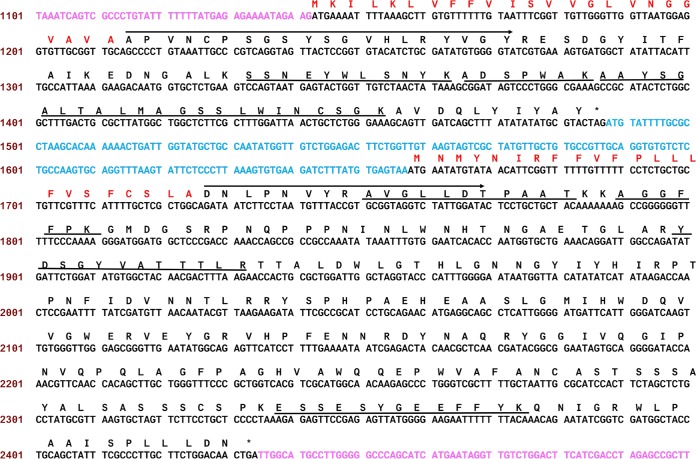
DNA and protein sequences of Pp-ANP1a/2a in the 2.6 kb DNA fragment. Dark DNA segments are Pp-ANP1a and Pp-ANP2a. Arrow lines above amino acid residues show the N-terminal peptide sequences and lines under amino acid residues indicate internal peptide sequences obtained with Edman degradation. Predicted signal peptides (amino acid sequences) are in red. Gap DNA sequence between two ORFs is in blue. Upstream and downstream DNA is in purple. Asterisks (*) indicate stop codons.

Based on the sequence information of this DNA fragment, specific primers were designed and a Genomic Walking kit was used to obtain the upstream and downstream sequences. After PCR, cloning, sequencing, and assembling, a 2.6-kb DNA sequence was obtained. This DNA contains two open reading frames (ORFs) in the same direction. The first ORF encodes a protein with 114 amino acids with a calculated molecular mass of 12.3 kDa. It contains the N terminus and all three internal peptide sequences from Peak-1 of RP-HPLC (Fig. 3 and Table 2). Upstream from the N-terminal peptide are 24 amino acids. Signal prediction software, SignalP, predicted that the first 24 amino acids comprise a secretion signal peptide. The calculated molecular mass of the mature protein is 9.8 kDa. The second ORF encodes a protein of 258 amino acids with a calculated molecular mass of 28.7 kDa, containing the N terminus and all four internal peptide sequences obtained from the Peak-2 sample (Fig. 3, Table 2). It also has a signal peptide in the N-terminal sequence. The calculated molecular mass of the mature protein is 25.9 kDa. Therefore, the two ORFs in the same orientation correspond to the Peak-1 and Peak-2 proteins, respectively. They were named Pp-ANP1a (KY945993) and Pp-ANP2a (KY945994) (the letter “a” was inserted because we identified homologous sequences from other P. protegens strains; see below). The two ORFs are separated by a 170-bp nontranslated sequence (Fig. 3). Neither gene product had a molecular mass that matched the 60-kDa value predicted based on elution in size exclusion chromatography. This is likely due to the multimerization of the two subunits prior to the final HPLC step.

A BLAST search of Pp-ANP1a did not result in a significant match to any known proteins in the NCBI and Swiss-Prot databases, while Pp-ANP2a showed ∼30% identity (∼40% similarity) to subunit A of Escherichia coli heat labile enterotoxin (LT) and cholera toxin (CT) (12, 13). After the elucidation of the 2.6-kb DNA sequence, more gene-specific primers were designed to walk further in both directions along the genome. A 6,378-bp sequence has been assembled. Only one portion downstream of Pp-ANP2a showed high sequence homology to part of the complete P. protegens Pf-5 genome (14). The whole genome of strain 15G2 was subsequently sequenced and the *Pp-ANP* sequence within the genome was further validated.

### Both proteins are required for the anti-nematode activity.

**FIG 4:**
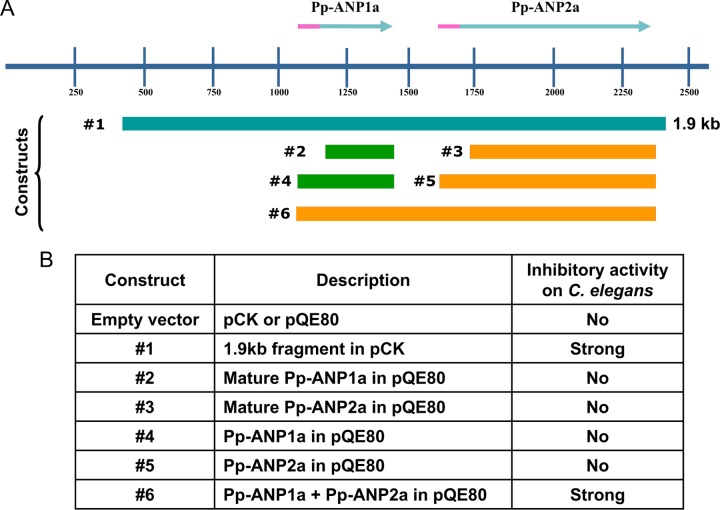
Cloning constructs and assay results. (A) Diagram of Pp-ANP DNA, Pp-ANP1a/2a and constructs tested. (B) Description of constructs and their activities on C. elegans.

**FIG 5:**
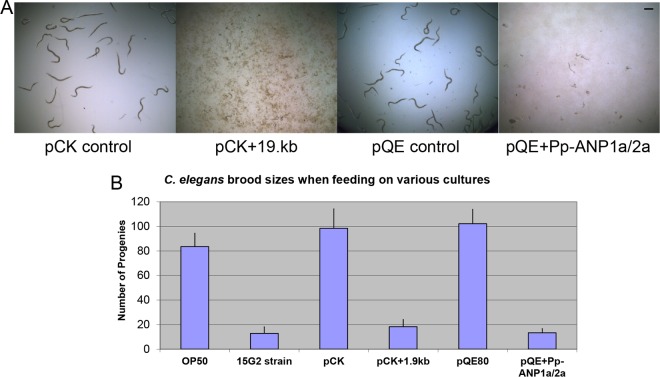
C. elegans assay with Pp-ANP expressed in E. coli. (A) C. elegans fed on E. coli containing empty plasmid pCK, Pp-ANP fragment in pCK (pCK + 1.9kb, Construct 1 in Fig. 4), empty plasmid pQE, or two Pp-ANPs in pQE (pQE + Pp-ANP1a/2a, Construct 6 in Fig. 4). (B) C. elegans average brood sizes with error bars after incubating single L4 worms in different cells for 5 days. Bar = 250 μm.

To confirm if the two ORFs are needed for the anti-nematode activity, we first subcloned a 1.9-kb fragment containing both Pp-ANP1a and Pp-ANP2a into E. coli. Feeding assays revealed that the transformed E. coli cells showed similar potency against C. elegans as the original 15G2 strain (Fig. 4 and Fig. 5A). This indicates that the 1.9-kb DNA fragment contains all components needed for the anti-nematode activity of the 15G2 strain. We further subcloned the two Pp-ANP components into the expression vector pQE80 individually. For each ORF, two constructs were made, one without the putative N-terminal signal peptide and the other with the signal peptide. We also made a construct containing both ORFs plus the spacer sequence between them (Fig. 4). In the C. elegans assay with isopropyl-β-d-thiogalactopyranoside (IPTG)-induced cell culture, none of the individual ORFs alone, with or without the signal peptide, showed activity under test conditions. However, the constructs containing both ORFs resulted in strong activity on C. elegans (Fig. 4 and 5A), indicating that both ORFs are required for the toxicity.

When tested with L1-stage C. elegans, both P. protegens 15G2 and E. coli expressing Pp-ANP showed inhibiting activity and arrested worms at the L1 stage. Arrested worms stayed alive for several days and likely developed into the dauer pathway, an arrested development stage that emerges when environmental conditions are unfavorable (15, 16). When L4-stage worms were fed E. coli expressing Pp-ANP toxin on agar plates, worms became very sick, moved very slowly, were severely damaged, and died within 24 h. Single L4 worms were transferred into 96-well titer plates, fed with either the original 15G2 strain or E. coli expressing the Pp-ANP toxins, and brood sizes were counted after 5 days. Worms feeding on OP50 and empty vector controls produced 83 to 102 progenies, while worms exposed to Pp-ANP toxin had significantly lower brood sizes with 12 to 18 progenies on average (Fig. 5B), all arrested at L1 stage.

### The two proteins can be reconstituted to form an active complex.

**FIG 6:**
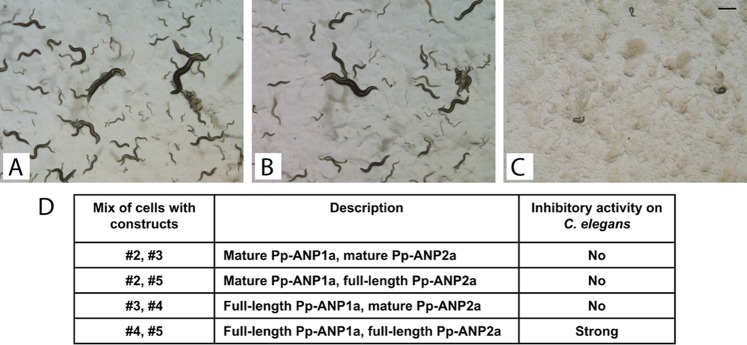
C. elegans assay with mixed E. coli cultures expressing individual Pp-ANP1a and PP-ANP2a. Images show worms growing on agar plates 3 days after seeding with L1 larvae. (A) Plate containing E. coli cells transformed with empty vector pQE80. (B) Plate containing mixed E. coli cells transformed with construct 2 and 3. (C) Plate containing mixed E. coli cells transformed with constructs 4 and 5. (D) Description of cell mixtures and their activities on C. elegans. Construct numbers are illustrated in Fig. 4. Bar = 250 μm.

We then tested the mixture of two types of E. coli cells expressing either of the two ORFs on C. elegans. The mixture of cells expressing full-length Pp-ANP1a and cells expressing full-length Pp-ANP2a showed inhibitory activity that is only a little weaker than cells expressing both ORFs together (Fig. 6). The cultures containing only mature proteins did not show any activity, indicating that the signal peptides are needed to express functional proteins. These results also indicate that the two proteins can be expressed separately and their activity can be reconstituted, apparently within the gut of the nematode.

Furthermore, we compared the effects of 6×His tags on protein activity. Constructs with N- or C-terminal 6×His tags were generated for both proteins. E. coli bacteria expressing Pp-ANP1a (N- or C-terminal 6×His) were mixed with the E. coli bacteria expressing Pp-ANP2a (N- or C-terminal 6×His), respectively, and assayed against C. elegans. Pp-ANP2a with the 6×His tag on either end behaved similarly, while Pp-ANP1a with C-terminal 6×His tag did not reconstitute the nematicidal activity when mixed with Pp-ANP2a. This indicates that C terminus of Pp-ANP1a is critical for its function and is sensitive to modifications.

### Pp-ANP is toxic to multiple nematode species.

**FIG 7:**
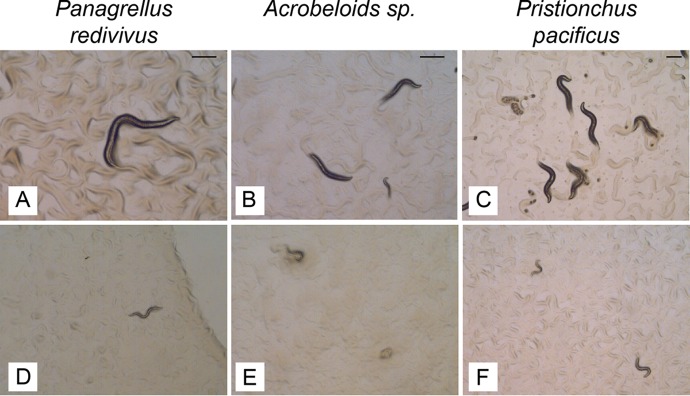
Pp-ANP1a/2a is toxic to other free-living nematodes. (A, B, C): E. coli transformed with empty vector; (D, E, F): E. coli expressing Pp-ANP1a/2a (construct 6 in Fig. 4). Bar = 250 μm.

We further tested the effects of Pp-ANPs on other free living nematodes (Pristionchus pacificus, Panagrellus redivivus, and Acrobeloides sp.) on agar plates with E. coli expressing Pp-ANP proteins. Pp-ANP clearly showed strong activity on all three nematode species by inhibiting worm growth and development (Fig. 7).

15G2 culture, *Pp-ANP*-transformed E. coli culture and its clear lysate were further tested on other targets, including insects (Helicoverpa zea, Diabratica virgifera, and Lygus hesperus) and fungi (Colletotrichum graminicola and Fusarium verticilliodes). No detrimental effects were observed on these organisms under test conditions. This indicates that Pp-ANP is not a general toxin, but shows selectivity toward nematodes.

### Pp-ANP homologous sequences are identified in other P. protegens strains.

Genome sequencing and protein purification of other C. elegans active strains of P. protegens in the same collection (11) indicate that strains 15H3 and 38G2 contain identical sequences for Pp-ANP1a and Pp-ANP2a, while strain 14B2 contains homologous sequences. The two proteins from strain 14B2 are named Pp-ANP1b (KY945995) and Pp-ANP2b (KY945996). Pp-ANP1b shows 84.2% sequence identity to Pp-ANP1a and Pp-ANP2b shows 93.8% identity to Pp-ANP2a. E. coli-expressing Pp-ANP1b/2b also showed nematicidal activity on C. elegans that was similar to but slightly weaker than that seen with Pp-ANP1a/2a. Further sequence mining identified another Pp-ANP homolog from P. protegens strain JH67972-2 from DuPont Pioneer's internal genome databases. The proteins from JH67972-2 are named Pp-ANP1c (KY945997) and Pp-ANP2c (KY945998). Pp-ANP1c and Pp-ANP2c show 85.1% and 95.0% identity to Pp-ANP1a and Pp-ANP2a, respectively. Pp-ANP1b/2b and Pp-ANP1c/2c share about 99% sequence identity for both subunits. No other homologous sequences were detected from the public databases, which contain multiple Pseudomonas genomes. The rare presence of Pp-ANP genes in Pseudomonas and other genomes indicates that Pp-ANPs are narrowly distributed.

### Pp-ANP2a is structurally similar to ADP-ribosyltransferases.

**FIG 8:**
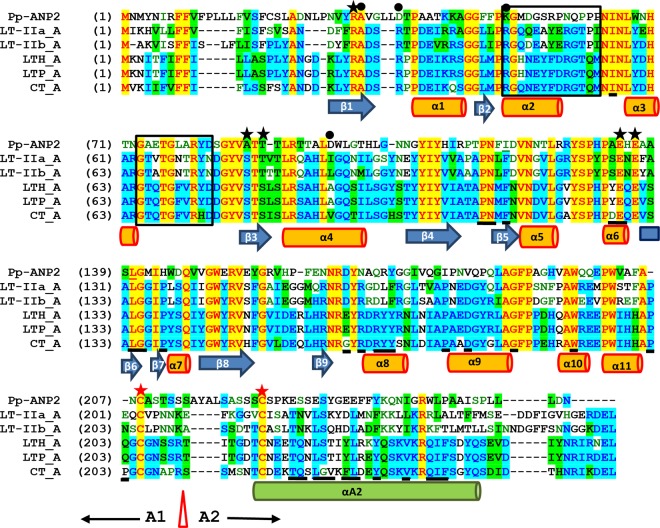
Protein sequence alignment of Pp-ANP2a and subunit A of CT/LT toxins. Blue arrow bars indicate β-sheet regions and orange cylinders α helical regions. Two boxes indicate the two activation loops. Red stars show the conserved cysteines and the red triangle shows cleavage sites in CT and LT toxins that cleave the protein into domains A1 and A2. Green cylinder shows the α helix in domain A2. Black stars highlight the highly conserved catalytic motifs (R/H-STS-ExE) across the ADPRT superfamily while black dots indicate additional NAD interacting residues. Underlined letters in the CTA sequence indicate A1 and A2 interface residues in the nonreduced/disulfide-bonded CTA form.

**FIG 9:**
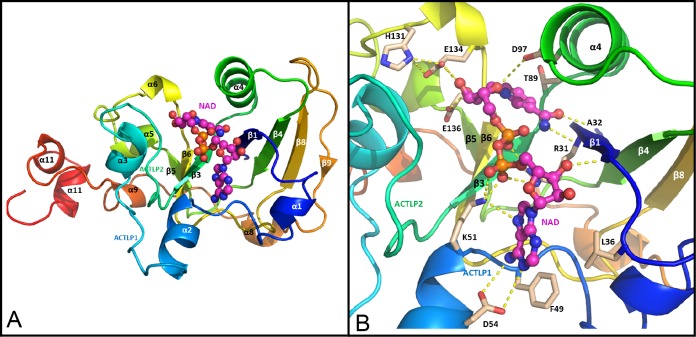
A Pp-ANP2a structural model built on the basis of a CTA1-ARF complex (PDB: 2A5F). (A) A cartoon representation of the overall model with NAD docked into the active site. ACTLP1 is activation loop 1 (51 to 63) and ACTLP2 is activation loop 2 (73 to 82). (B) Detailed NAD binding mode. The yellow dash-lines indicate hydrogen bonds.

Since Pp-ANP2a shows ∼30% sequence identity to CT/LT toxins, we further analyzed the sequence and structural similarity. We built a model of Pp-ANP2a based on the structure of cholera toxin subunit A (CTA) (17). The model is highly similar to CTA1 in all major secondary structure elements and the structural core, including the central β-sheet and catalytic residues (Fig. 8). It has the highly conserved typical ADP-ribosyltransferase (ADPRT) α/β fold with a central 7-stranded anti-parallel β-sheet surrounded by helices. The central β-sheet is drastically twisted at middle with subsheet β9-β8-β4-β1 roughly perpendicular to subsheet β3-β6-β5. Consequently, the perpendicular neighboring strands β1 and β3 form a cleft, the putative active site harboring NAD (Fig. 9A). Consistent with known arginine ADPRTs, the bound NAD is forced to assume an energetically unfavorable conformation in which its nicotinamide ring is sandwiched between β3, with its conserved S-T-S motif, and α4, supporting the so-called “strain-alleviation” SN1-like catalytic mechanism (18). Compared to CTA, Pp-ANP2a replaces the S-T-S motif in β3 with A^87^-T-T^89^ and substitutes another NMN-ring-packing residue, Val in α4 in CTA, with Asp^97^. These changes, however, largely preserve the binding geometry. Pp-ANP2a also possesses crucial catalytic motifs (R^31^-ATT^89^-ExE^136^) (Fig. 8 and 9B) corresponding to the hallmark pattern of ADPRTs (R/H-STS-ExE).

The similarity of the Pp-ANP2a model to the CTA structure in secondary structural elements and in the placement of amino acids associated with CTA's catalytic mechanism strongly suggests that Pp-ANP2a functions as a CTA-like phosphoribosyl transferase. However, there are differences that differentiate Pp-ANP2a from known ADPRTs. (i) Compared to CTA, the ADP adenine binding loop spanning β1 and α1 lacks the corresponding arginine that forms the Arg-π bond with the adenine base in CTA, but instead has a two-leucine (Leu^35^ and Leu^36^) insertion (Fig. 8). (ii) Although activation loop 2 (G^73^ to D^82^) is well conserved, loop K^51^to P^63^ in Pp-ANP2a has almost no homology to its counterparts, known as activation loop 1 in other ADPRTs. (iii) There is significant divergence in Pp-ANP2a in the region C^208^ to C^225^, which in other ADPRTs comprises the A1-A2 SS-bond linkage. In contrast to CTA, Pp-ANP2a lacks either a lysine or arginine, which in CTA confers a trypsin cleavage site. Furthermore, the amino acids identified as belonging to the A1-A2 interface in CTA (Fig. 8, underlined residues) are quite divergent in Pp-ANP2a. (iv) Finally and very significantly, Pp-ANP2a lacks the KDEL or RDEL endoplasmic reticulum (ER) retention signal present in all other CT and LT toxins. All these obvious sequence divergences indicate that Pp-ANP2a might have a different activation/action mechanism.

## DISCUSSION

A number of Pseudomonas strains display nematicidal activity on free-living or parasitic nematodes and have been explored as potential biocontrol agents. The root-colonizing bacterium P. fluorescens CHA0, which was isolated from a suppressive soil, has been studied in detail as a strain for the biological control of root-knot nematodes (19, 20). It excretes multiple antimicrobial compounds, such as 2,4-diacetylphloroglucinol, pyoluteorin, pyrrolnitrin, and hydrogen cyanide. The production of the major extracellular EDTA-sensitive protease AprA also contributes to the detrimental effects on root-knot nematodes (21). A proteinaceous insect toxin, Fit, has also been identified from P. fluorescens strains CHA0 and Pf-5 (22). The Fit protein is related to insect toxin Mcf1 of the entomopathogen P. luminescens. P. aeruginosa strains kill C. elegans by diffusible toxins and infection, and are able to suppress the expression of a subset of immune defense genes in C. elegans by activating the DAF-2/DAF-16 insulin-like signaling pathway (2, 23). P. brassicacearum strain DF41 is capable of killing C. elegans through toxic metabolites and biofilm formation on the nematode head blocking the buccal cavity (24). Some Pseudomonas bacteria produce exotoxins, which are toxic to the larvae of mosquitoes and house flies by inhibition of protein synthesis (25, 26). P. aeruginosa exotoxin ToxA does not affect C. elegans life span but triggers the worm immune response (27). The Pp-ANPs described in this study are different from any of the previously reported Pseudomonas toxins.

**FIG 10:**
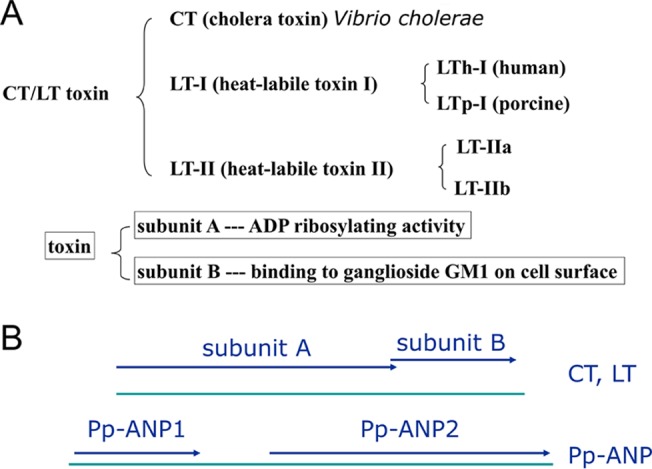
CT/LT toxins and gene organization. (A) CT/LT toxin types. (B) Comparison of gene organization of enterotoxin and Pp-ANP.

Heat-labile enterotoxins belong to the CT-LT family of AB5 toxins (12, 13, 28). CT is produced from Vibrio cholerae O1 and LT is from enterotoxigenic E. coli. CT and LT share structural and functional similarity. Both toxins are composed of one A subunit which has ADP-ribosylation activity and five B subunits which recognize and bind to ganglioside GM1 on eukaryotic cell surfaces. There are two serotype LT toxins, LT-I and LT-II (Fig. 10A). LT-I includes subtype LTh-I, which causes generally milder “traveler's” diarrhea of human, and LTp-I, which is only found in porcine isolates. The second group of E. coli heat-labile enterotoxins, LT-II, contains LT-IIa and LT-IIb (13). Pp-ANP2a showed the highest homology (42% similarity, 30.1% identity) to LT-IIa subunit A. However, Pp-ANP1a shows very low homology (25.8% similarity, 12.9% identity) to LT-IIa B subunit. It is likely that the holotoxin of Pp-ANP is a heteromultimeric AB toxin, as the molecular mass we observed for the active protein from P. protegens strain 15G2 is about 60 kDa. Our model strongly indicates that Pp-ANP2a is highly conserved with CTA1 in secondary and tertiary structure, and likely acts as a typical ADP-ribosyltransferase.

Further analysis reveals that Pp-ANP is distinct from CT/LT toxins in several characteristics. First, the gene organization is different. The large (A) subunit is upstream of subunit B in CT and LT toxins and the two ORFs have some overlapping DNA sequence. In Pp-ANP, the small subunit (Pp-ANP1a) is upstream of the large subunit (Pp-ANP2a) and there is a 170 bp gap between the two ORFs (Fig. 10B and 3). Second, all CT/LT toxins contain either KDEL or RDEL, the ER retention signals at the C terminus of the subunit A (Fig. 8). Experimental evidence indicated that CT and LT interact directly with endogenous KDEL-receptors and that the ER retention signal is important for efficient and maximal biologic activity of CT and LT in the target organisms (12). Pp-ANP2a does not have an ER retention signal in the C terminus, suggesting that Pp-ANP targets a different cell compartment than CT and LT toxins. Third, the two well-conserved cysteines in enterotoxin subunit A form a disulfide bond covalently connecting the A1 and A2 domains (29). The cleavage site between A1 and A2 for CT/LT toxins is after the arginine or lysine, but there is no arginine or lysine residue between the two cysteines in Pp-ANP2a (Fig. 8). Finally and more importantly, the A1 domain in CT/LT toxin is solely responsible for the toxin's enzymatic activity, while the A2 domain is responsible for tethering the A1 domain to the pentameric B subunit, which binds to apical membrane receptors (30). Therefore, the A2 domain and the B subunit together in CT/LT toxins contribute cell surface targeting and binding properties. However, A2 and subunit B sequences do not share significant similarity with the C-terminal region of Pp-ANP2a (Fig. 8) and Pp-ANP1a, respectively. Nor are most of the amino acids in CTA that constitute the interface between CTA1 and CTA2 conserved in Pp-ANP2a (Fig. 8). The absence of a trypsin cleavage site in Pp-ANP2a leads us to speculate that proteolytic cleavage and disulfide bond reduction are not required for activation of Pp-ANP2a as they are in CT. The notion is further supported by the observation that pP-ANP2a has serine at position 56. A CTA mutant in which tyrosine at the same contextual position is mutated to serine is characterized as requiring no modification for full activity (31). Interestingly, the C-terminal end of Pp-ANP2a has a highly hydrophobic stretch, WLPAAISPLLL, which could conceivably comprise an element for interaction with Pp-ANP1a.

The data in the present study show that Pp-ANP possessed strong killing activity toward various nematodes, but no activity on insects and fungi. It has been reported that the lethal infection of V. cholerae in C. elegans is caused by extracellular protease PrtV or hemolysin HlyA, but not by CT toxin (32, 33) and that E. coli LT toxin only shows weak activity on C. elegans (34). This present study reports an enterotoxin-like protein from a Pseudomonas species and its strong anti-nematode activity. Though it needs further investigation, we propose a hypothetical model in which Pp-ANP acts similarly to an AB5 toxin. Briefly, Pp-ANP is a complex of one ANP2a subunit and (perhaps) five ANP1a subunits. The whole complex binds specifically to receptors on nematode gut cells via the ANP1a pentameric structure; the toxin enters the targeted cells through endocytosis and stays in the cytosol since Pp-ANP2a lacks a C-terminal KDEL/RDEL signal to transport it to the ER, as CTs/LTs normally do. Then the Pp-ANP2a performs ADP-ribosylation, which causes a series of downstream molecular and cellular events and the damaged gut cells eventually cause nematode growth inhibition and death. Overall, Pp-ANP represents a new member of the heat-labile enterotoxins. Its specific target binding and cellular integration make it a unique member of this family and imply a new mechanism of action.

The virulence genes for LT of enterotoxigenic E. coli are carried by plasmids, whereas the gene for CT of V. cholerae O1 is on the chromosome, introduced by horizontal gene transfer from a lysogenic bacteriophage (35). The genome sequence indicates that *Pp-ANP* is located on the chromosome of strain 15G2, but is not present in other P. protegens genomes available in the public databases (14, 36), even though the 16S sequences of these strains are identical. The real origin of the Pp-ANP awaits further investigation. It is interesting to note that there is a short fragment (126 bp) upstream near the *Pp-ANP* 5′ end homologous to some Pseudomonas plasmid sequences, which implies the *Pp-ANP* could be obtained by gene integration from a plasmid during evolution.

Pp-ANPs have potential applications in controlling parasitic nematodes. Only a few anti-nematode proteins have been reported (37). Certain lectins can enhance plant defense against pathogens and nematodes (38, 39). Some Cry proteins from Bacillus thuringiensis are active against diverse nematodes (40, 41). Cry6A and Cry5B genes have been tested in transgenic plants and showed partial control of root-knot nematodes (42, 43). Plant-parasitic nematodes, such as cyst nematodes and root-knot nematodes have evolved unique feeding apparatuses, stylets, and feeding tubes which exclude large molecules from being taken up. It has been shown that molecules larger than 40 kDa cannot be efficiently delivered into cyst nematodes (44). The individual Pp-ANP1a and Pp-ANP2a are smaller than that limit and can be reconstituted into functional units. It may be possible for the two proteins to be expressed and delivered separately and form the active multisubunit protein inside the nematode gut. Our preliminary tests indicated that Pp-ANP1a and Pp-ANP2a can be expressed individually in tobacco leaves by coinfiltration and form active protein (data not shown). However, formation of toxin by strong association prior to uptake could present an engineering challenge. Other plant-parasitic nematodes, such as lesion or reniform nematodes that lack feeding tubes, may not have such exclusion sizes and could be easier targets of Pp-ANP. Human- and animal-parasitic nematodes have adapted complex life cycles, which include free-living stages and parasitic stages (45). The free-living-stage larvae feed on bacteria and are sensitive to some bacterial toxins (41). Cry5B protein showed good efficacy to treat some of those nematodes (46). Pp-ANP also has potential in applications for controlling the free-living stages of human- and animal-parasitic nematodes. Furthermore, C. elegans has been used as a model system to study host-pathogen interactions (2, 47). Pp-ANP identified in this study is different from other known nematicidal toxins. It can be used to study host-toxin interactions and the evolutionary relationships between nematodes and anti-nematode microbes.

## MATERIALS AND METHODS

### Nematodes and bacterial strains.

Caenorhabditis elegans N2, Pristionchus pacificus PS312WT, Panagrellus redivivus MT8872, and Acrobeloides sp. DWF1105 were ordered from the Caenorhabditis Genetics Center (CGC, https://cbs.umn.edu/cgc/). Worms were maintained at room temperature on nematode growth media (NGM) agar plates supplemented with Escherichia coli strain OP50 as a food source. Multiple bacterial strains were obtained from soil samples collected throughout the United States, including samples from Missouri, Mississippi, and Wyoming (11). The strains were grown in King's medium at 30°C with shaking at 225 rpm overnight.

### Anti-nematode assay.

C. elegans L1 larvae were synchronized by bleaching adult worms and hatching eggs overnight (48). Anti-nematode assays were carried out in 96-well microtiter plates. Each assay well contained ∼50 L1 staged C. elegans, 30 μg/ml tetracycline, 30 μg/ml chloramphenicol, E. coli OP50 at 1/10 the density of a saturated culture, and 5 to 30 μl of the overnight culture or protein samples to be tested in 120 μl of S-medium (40, 41). In negative controls, E. coli OP50 was used as the only food source. Forty-eight hours later, the assay plates were scored microscopically by visually rating the worms' growth and development. For the brood size test, individual L4 worms were picked from NGM agar plates and transferred into 96-well microtiter plates, with a single worm per well. Each well contained 120 μl of the same medium as for the anti-nematode assay. After incubation at room temperature for 5 days, numbers of progeny in each well were counted. For the anti-nematode assay on P. pacificus, P. redivivus, and Acrobeloides sp., L1 stages nematodes were added to recombinant E. coli lawn on agar plates. After 4 days and 8 days, the growth was scored.

### Protein separation and purification.

Nematicidal activity was tracked with the C. elegans bioassay described above. Column fractions were assayed with a single volume of each fraction, while for pooled fractions a dose response was obtained. A unit of activity is the volume resulting in 80% growth inhibition (GI80). Total activity in a fraction is the total number of GI80 doses (total volume of the fraction/GI80 dose volume). Protein concentration was determined by the Bradford method using bovine serum albumin (BSA) as standard. P. protegens 15G2 was grown at 30°C for 20 h in 1-liter flasks containing 250 ml King's medium. Cultures were spun at 10,000 × *g* for 10 min, and cell pellets were stored at −80°C. Cells were resuspended in a total of 30 ml of 50 mM HEPES, pH 7.2, 10 mM potassium phosphate, 100 mM KCl, 5% ethylene glycol, 1 mM dithiothreitol (DTT), and protease inhibitor cocktail (2 mg/ml, Sigma-Aldrich) and lysed by extrusion from a French Pressure Cell Disruptor (Thermo Scientific) at 18,000 lb/in^2^. The lysate was sonicated to reduce the viscosity and spun at 100,000 × *g* for 30 min. The supernatant was passed through a 0.2-μm filter, desalted by gel filtration through Sephadex G25 equilibrated with 50 mM HEPES, pH 7.2, 20 mM KCl, and 5% ethylene glycol (G25 buffer), and passed through an anion exchange resin (HiPrep Q, 16/10, GE Health Science) equilibrated with G25 buffer. The nematicidal activity was present in the flowthrough. Turbidity was removed by centrifugation at 100,000 × *g*.

The HiPrep Q flowthrough (∼50 ml) was concentrated 2- to 3-fold by ultrafiltration (Amicon Ultra-15, YM 30 membrane), then gel-filtered into sulfopropyl start buffer (25 mM HEPES, pH 6.9 and 5% ethylene glycol). This material was then applied to HiPrep sulfopropyl cation exchange resin (16/10, bed vol 20 ml; GE Health Sciences) equilibrated with start buffer, and was eluted with a linear gradient to 200 mM KCl in sulfopropyl start buffer. Active fractions were pooled, concentrated (YM30), buffer exchanged into 25 mM HEPES, pH 6.9, then chromatographed on a high-resolution cation exchanger, CM825 (Tosoh). The anti-nematode activity eluted within a shallow gradient of 160 to 200 mM KCl in 25 mM HEPES, pH 6.9.

Reverse phase HPLC was carried out with a Jupiter C5 column (150 × 2 mm, Phenomenex) with a gradient of acetonitrile from 20% to 60% in 40 min, while 0.2% trifluoroacetic acid declined from 14% to 10%. The candidate proteins were transferred from SDS-PAGE gel to a polyvinylidene difluoride (PVDF) membrane and protein bands were submitted for N-terminal sequencing with Edman degradation.

### PCR and cloning.

Genomic DNA was extracted from an overnight culture with Sigma Bacterial DNA Extraction kit (Sigma-Aldrich). Degenerate primers were designed based on N-terminal and internal amino acid sequencing data. PCR was carried out at 94°C for 2 min; 25 cycles of 94°C for 15 s, 52°C for 30 s, and 72°C for 2 min; and 72°C for 10 min. PCR products were excised from agarose gel and eluted from gel slices with a Qiagen gel purification kit. The DNA was then cloned into TOPO vector with T/A cloning kit (Invitrogen) and the inserts were sequenced. The Genome Walker Universal kit from Clontech was then used to obtain upstream and downstream genomic DNA sequences. The DNA fragment containing Pp-ANP was subcloned into an internal vector pCK and predicted open reading frames (ORFs) were subcloned into vector pQE80 (Qiagen). The recombinant proteins were expressed in E. coli under IPTG induction and expression detected with SDS-PAGE. E. coli cultures containing recombinant proteins were fed to C. elegans as described above.

### Structure modeling.

CTA-NAD/ARF-GTP complex structure (PDB: 2A5F) was selected as modeling template (17) because it shows a decent similarity in pairwise comparison and it also fully exhibits NAD binding mode in details. Based on the target-template alignment, 20 different three-dimensional (3D) models were generated by MODELER 9.15 in Discovery Studio (DS; Accelrys Software Inc., BIOVIA 2016 release) and the model with the lowest discrete optimized protein energy (DOPE) score was used for NAD docking and manually adjusted at a loop spanning α1 and β1 with a two-Leu insertion. The quality of the model was verified with MODELER, which yielded a normalized DOPE score of <−0.62 and showed that all side chains resided in the allowed area in the Ramachandran plot.

### Accession number(s).

The sequences of Pp-ANP have been submitted to GenBank under the accession numbers KY945993 to KY945998.
